# 1578. Evaluation of comparative HIV care continuum outcomes following three years of program implementation of the PositiveLinks smartphone intervention among people with HIV with and without substance use

**DOI:** 10.1093/ofid/ofad500.1413

**Published:** 2023-11-27

**Authors:** Jacqueline Hodges, Sylvia Caldwell, Kori Otero, Brooke Williams, Ben Elliott, Lauren Richey, Ava Lena Waldman, Rebecca Dillingham, Karen Ingersoll

**Affiliations:** University of Virginia, Charlottesville, Virginia; University of Virginia, Charlottesville, Virginia; University of Virginia, Charlottesville, Virginia; University of Virginia, Charlottesville, Virginia; University of Virginia, Charlottesville, Virginia; LSU Health Sciences Center New Orleans, New Orleans, Louisiana; University of Virginia, Charlottesville, Virginia; University of Virginia, Charlottesville, Virginia; University of Virginia, Charlottesville, Virginia

## Abstract

**Background:**

People with HIV (PWH) and substance use (PWSU) have demonstrated inferior HIV care continuum outcomes, including lower retention in care (RIC), poorer ART adherence, and lower rates of viral suppression (VS) relative to peers without substance use within the literature. We compared 3-year outcomes among people with and without substance use who are enrolled in a smartphone intervention, PositiveLinks (PL), to determine the relative impact of PL for this subgroup of PWH.

**Methods:**

We performed retrospective chart review of clinical encounter and laboratory data and ICD-diagnoses for 600 PWH enrolled in PL from 2017-2020. Substance use was categorized by diagnoses of illicit substance use and/or substance use-related visits conducted at the UVA Ryan White Clinic. Patients with no documented substance use, or documented tobacco or reported alcohol use with no criteria for a substance use disorder were considered non-substance using. We analyzed HIV clinical outcomes (VS, RIC by HRSA-1 measure) for PL members in each group at baseline (April 2017) and three years later (April 2020, t-test).

**Results:**

Chart review for the 3-year period of interest for 600 PWH enrolled in PL revealed 117 PWH meeting criteria for substance use and 483 not meeting criteria. A mean of 13 Ryan White Clinic visits were conducted per person among 117 PWSU, compared to 8 visits each among 483 PWH without documented substance use (p< .01) over 3 years. Among PWSU, RIC increased from 56% to 79% (*p*< .01) while among PWH without substance use, RIC increased from 47% to 69% (*p*< .01). VS increased significantly among PWSU from 71% to 90% (p< .01), while among PWH without documented substance use, VS increased from 70% to 87%, (p< .01). Differences between groups for RIC and VS were not statistically significant at either time point.

**Conclusion:**

A sample of 600 PWH using PL demonstrated comparable HIV clinical outcomes across 3 years regardless of substance use status. This sample selects for PWSU who stayed enrolled in PL at 3 years, and may not reflect outcomes expected for a broader population of variably engaged PWSU offered non-tailored mobile health interventions for HIV care. Further analysis is planned to determine whether covariates including demographic characteristics and PL usage patterns influence outcomes observed.

**Disclosures:**

**Sylvia Caldwell, MPH, DHSc**, Warm Health Technology: Advisor/Consultant **Ben Elliott, MSW**, Warm Health Technology: Advisor/Consultant **Ava Lena Waldman, M.H.S., CHES, CCRP**, Warm Health Technology: Advisor/Consultant **Rebecca Dillingham, MD MPH**, Warm Health Technology: Advisor/Consultant

